# Hydrothermal treatment and butylphosphonic acid derived self-assembled monolayers for improving the surface chemistry and corrosion resistance of AZ61 magnesium alloy

**DOI:** 10.1038/s41598-017-17199-z

**Published:** 2017-12-04

**Authors:** Chung-Wei Yang, Cheng Liu, Da-Jun Lin, Ming-Long Yeh, Tzer-Min Lee

**Affiliations:** 1 Department of Materials Science and Engineering, National Formosa University, Yunlin, Taiwan; 2 Hyperbaric Oxygen Therapy Center and Division of Plastic Surgery, Chi Mei Medical center, Tainan, Taiwan; 3 Department of Electrical Engineering, Southern Taiwan University of Science and Technology, Tainan, Taiwan; 4 Department of Biomedical Engineering, National Cheng Kung University, Tainan, Taiwan; 5 Institute of Oral Medicine, National Cheng Kung University, Tainan, Taiwan; 6 School of Dentistry, National Cheng Kung University, Tainan, Taiwan; 7 Department of Dentistry, Kaohsiung Medical University Hospital, Kaohsiung, Taiwan

## Abstract

The hydrothermal treatment followed by a self-assembled monolayer (SAM) of 1-butylphosphonic acid through the tethering by aggregation and growth (T-BAG) method was employed to produce protective surface coatings on the Mg-6Al-1Zn alloy (AZ61) for reducing the degradation rate in physiological environments. Potentiodynamic polarization measurements revealed that the organic self-assembled monolayer and Mg(OH)_2_ coating can further enhance the surface chemical stability and corrosion resistance of Mg alloys. SAM-treated Mg(OH)_2_ coatings can be served as a more passive surface layer as a result of their much higher charge transfer resistance and the presence of Warburg impedance in electrochemical impedance spectroscopy measurement.

## Introduction

Mg-based alloys, which have the lowest density (1.74 g/cm^3^) of any metallic constructional materials, are widely used in automobiles and aerospace for the purpose of reducing vehicle weight and fuel consumption^1^. Mg alloys also have attracted extensive attention of nowadays as potential lightweight implants in biomedical applications due to their high specific strength (strength-to-weight ratio) and perfect biocompatibility^2–4^. Since the elastic modulus of Mg alloys (41–45 GPa) is closer to the cortical bone (3–20 GPa) and much lower than biocompatible titanium alloys (about 110 GPa)^5^, serious stress shielding effect^6^, which resulted from much higher elastic moduli of implants compared with the human bone tissues, can be further reduced during the bone healing process^7^. In addition, Mg^2+^ is the fourth most abundant cation in the human body, and Mg is regarded as a basic element in the growth of new bone tissue^8–10^. Relative studies also indicated that Mg is an essential element to metabolism and enzyme systems, and Mg^2+^ ions can stabilize the structures of DNA and mRNA^11–13^. Therefore, Mg and its alloys have been successfully applied as scaffolds, load bearing and biodegradable orthopedic implants in the physiological environment^2–5,14–17^. Several Mg alloys, such as Mg-Al-Zn, Mg-Al-Mn, LAE442, Al-free WE43, Mg-Zn and Mg-Ca alloys^4,5,18–21^, are investigated and developed for biodegradable metallic materials. However, Mg is chemically active in nature, and the applications of Mg alloys are usually limited due to their high corrosion rate. Several clinical studies have shown that Mg alloys undergo severe corrosion under physiological conditions^5,20–22^. Thus, the degradation rate of Mg alloys should be slowed down to allow the mechanical integrity of the implants to remain intact before the adequate healing of new tissues.

Surface modification, which includes chemical conversion coating, micro-arc oxidation, anodizing, electrochemical deposition and hydrothermal crystallization methods, is considered as an effective technique to improve the corrosion resistance of Mg alloys^23–27^. Magnesium hydroxide (Mg(OH)_2_), Mg-Al hydrotalcite and bioactive hydroxyapatite/fluorohydroxyapatite (HA/FHA) compounds are commonly applied as surface protecting coatings of Mg alloys^27–33^ due to their non-toxicity, excellent biocompatibility and bioactive properties to the human body. In addition, chemical modification of inorganic material substrates with organic compounds is another active research area in the fields of biotechnology. It is noted that the self-assembled monolayers (SAMs) technique can provide a well-defined surface chemistry of biomaterials and has been applied to investigate effects of organic functional groups on the nucleation and growth of calcium phosphate coatings on metallic substrates^34–36^. Methyl (–CH_3_), hydroxyl (–OH), carboxyl (–COOH) and amine-terminated (–NH_2_) functional end groups are commonly used terminal groups of SAMs. The Langmuir-Blodgett method^37^ and the tethering by aggregation and growth (T-BAG) method^38^ are two typical SAMs techniques to modify the surface chemistry of inorganic materials. The advantage of T-BAG method to prepare SAMs is that the aforementioned organic functional groups can be directly transverse to the substrates without promoting surface activation or applying of pressure. In the present study, a uniform magnesium hydroxide (Mg(OH)_2_) surface protective coating was deposited on the AZ61 Mg alloy by the hydrothermally synthesizing process based on our previous researches^31,33^. Following the hydrothermal treatment, a SAM surface treatment was performed by the T-BAG method in order to further improve the surface chemical stability of the Mg(OH)_2_-coated AZ61 Mg alloy.

The purpose of this study is to improve the surface chemical stability and enhance the corrosion resistance of biodegradable AZ61 alloy within physiological environments. The surface crystal structure and chemical states of the AZ61 alloy after the application of hydrothermal coating with SAM surface treatment were analyzed using grazing-incidence X-ray diffraction (GI-XRD) and X-ray photoelectron spectroscopy (XPS), respectively. The corrosion resistance of the surface coated AZ61 alloy was investigated in a standard simulated body fluid (R-SBF).

## Results and Discussion

**Figure 1:**
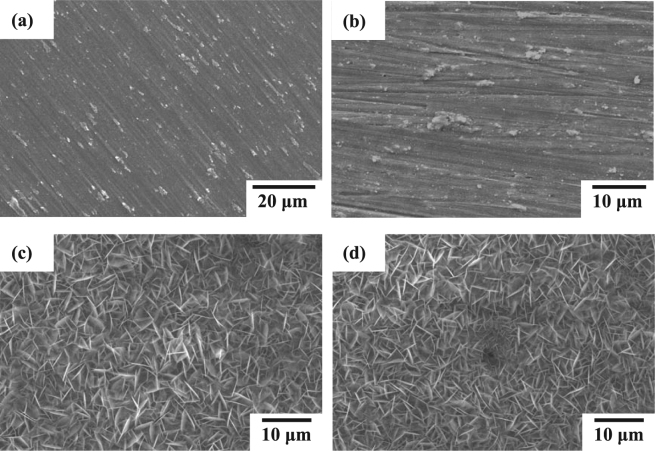
SEM surface morphologies of the (**a**) polished AZ61 Mg alloy, (**b**) alkaline pre-treated AZ61-AP, (**c**) hydrothermally treated AP-HT, and (**d**) T-BAG surface treated HT-SAM specimens.

Figure 1 shows the representative SEM surface micrographs of the uncoated (surface polished) AZ61 Mg alloy, alkaline pre-treated AZ61-AP, hydrothermally treated AP-HT and SAM-treated HT-SAM specimens. Compared with Fig. 1(a) and (b), there is no significant difference in the surface morphology between the polished AZ61 and the AZ61-AP specimens. It reveals that surface grooves are present on AZ61 alloy after completion of the polishing and alkaline pre-treating processes. After performing the hydrothermal treatment, we can see that a typical surface morphology with nanoscaled platelet-like shape compounds is observed on the surface of AP-HT specimens, as illustrated in Fig. 1(c). Since the magnesium hydroxide (Mg(OH)_2_) is a common corrosion product for Mg alloys immersed in aqueous environments, it can be recognized that a nanoscaled platelet-like shape Mg(OH)_2_ layer is uniformly deposited on AZ61 substrate by the hydrothermal crystallization process. Several reports indicated that surface morphologies with nanoscaled crystals could effectively improve the bioactivity and cellular biocompatibility of substrates^28,33,39^. Therefore, it can be expected that the hydrothermally crystallized Mg(OH)_2_ with microstructures illustrated in Fig. 1(c) will further assist in cells proliferation. Figure 1(d) shows the surface morphology of HT-SAM specimens, which displays the same microstructural feature as AP-HT specimens. As a result, it can be seen that the T-BAG SAM treatment by an organic phosphonic acid will not change the surface structure of hydrothermal crystallized Mg(OH)_2_ layer. In addition, the distribution of Mg, O and P elements on AP-HT and HT-SAM speimens is also analyzed by the EDS-mapping patterns. According to the analysis results, both of AP-HT and HT-SAM specimens display a uniform distribution of Mg and O elements on the surfaces. The detected Mg and O elements should be resulted from the surface nanoscaled platelet-like shape Mg(OH)_2_ layer. However, few of P element is detected on AP-HT and HT-SAM specimens by the EDS analysis. Since the deposited SAM only changes the surface chemistry of substrates, and relative study also indicated that the organic phosphonic acid derived SAMs is about 1 to 2 nm^40^. Therefore, P elements within the T-BAG SAM treated surface monolayer on HT-SAM specimens is hardly analyzed by the SEM/EDS analysis method. However, the chemical states of surface elements for Mg(OH)_2_-coated AP-HT specimens may be varied with performing the T-BAG SAM treatment, and the difference will be clarified by the XPS analysis results in the next section.

**Figure 2:**
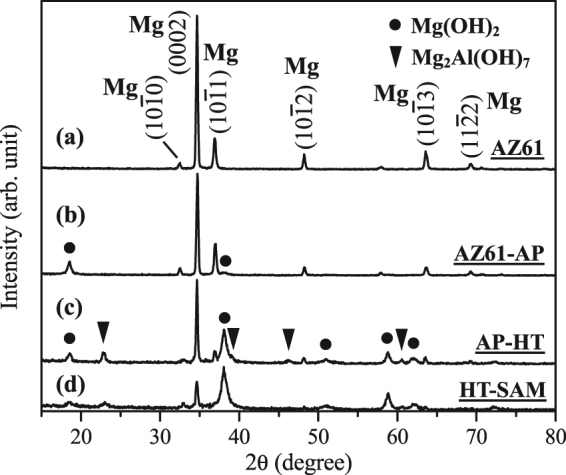
Grazing-incidence X-ray diffraction patterns of the AZ61 Mg alloy, AZ61-AP, AP-HT and HT-SAM.

Figure 2(a) shows the grazing-incidence X-ray diffraction (GI-XRD) patterns of the uncoated AZ61 Mg alloy. Compared with the standard powder diffraction of α-Mg (JCPDS 35-0821), the (0002) basal plane, which displays the strongest peak intensity, can be denoted as the preferred orientation of AZ61 Mg alloy. It is recognized that Mg alloys with preferred orientation of (0002) basal-textured orientation generally exhibited higher corrosion resistance in the physiological environments^41^. Figure 2(b) shows the GI-XRD pattern of alkaline pre-treated AZ61-AP specimens, which also included diffraction peaks of α-Mg substrate and Mg(OH)_2_ (brucite phase, main peaks detected at 2θ = 18.6° and 38.0°, JCPDS 07-0239). It represents that only a little amount of Mg(OH)_2_ formed on the AZ61 alloy after the alkaline solution pre-treatment. Figure 2(c) displays the diffraction pattern of hydrothermally treated AP-HT specimens. The obviously sharpening of Mg(OH)_2_ main peaks intensity means that the crystallinity and phase content of Mg(OH)_2_ is effectively improved after the hydrothermal treatment. It also demonstrates that a uniform nanoscaled platelet-like shape Mg(OH)_2_ thick layer (as shown in Fig. 1(c)) can be easily produced on AZ61 alloy through the hydrothermal crystallization process. In addition, a Mg_2_Al(OH)_7_ compound (diffraction peaks at 2θ = 23.3°, 39.3°, 46.8° and 60.5°, JCPDS 35-1275) is also observed in the surface coating of AP-HT specimens, as indicated by the triangular marks in Fig. 2(c). Mg_2_Al(OH)_7_ is a kind of hydrotalcite-like layered Mg/Al double hydroxide (LDH) compound with a hexagonal crystal structure derived from brucite Mg(OH)_2_, where Al^3+^ cations replace some of the Mg^2+^ cations^42^. As a result, it can be recognized that Mg_2_Al(OH)_7_ is another minor corrosion product accompanied with the formation of Mg(OH)_2_ for the AZ-series Mg alloys immersed in an aqueous environment. After the SAM surface treatment, X-ray diffraction peaks of α-Mg, brucite Mg(OH)_2_ and Mg_2_Al(OH)_7_ are detected in HT-SAM specimens, as illustrated in Fig. 2(d). The relative peaks intensity of Mg(OH)_2_ is increased. However, the peaks intensity of Mg_2_Al(OH)_7_ is reduced after SAM surface treatment, and it can be deduced that the decreased crystallinity of Mg_2_Al(OH)_7_ is resulted from the dehydration during the vacuum heating step of SAM treatment.

**Figure 3:**
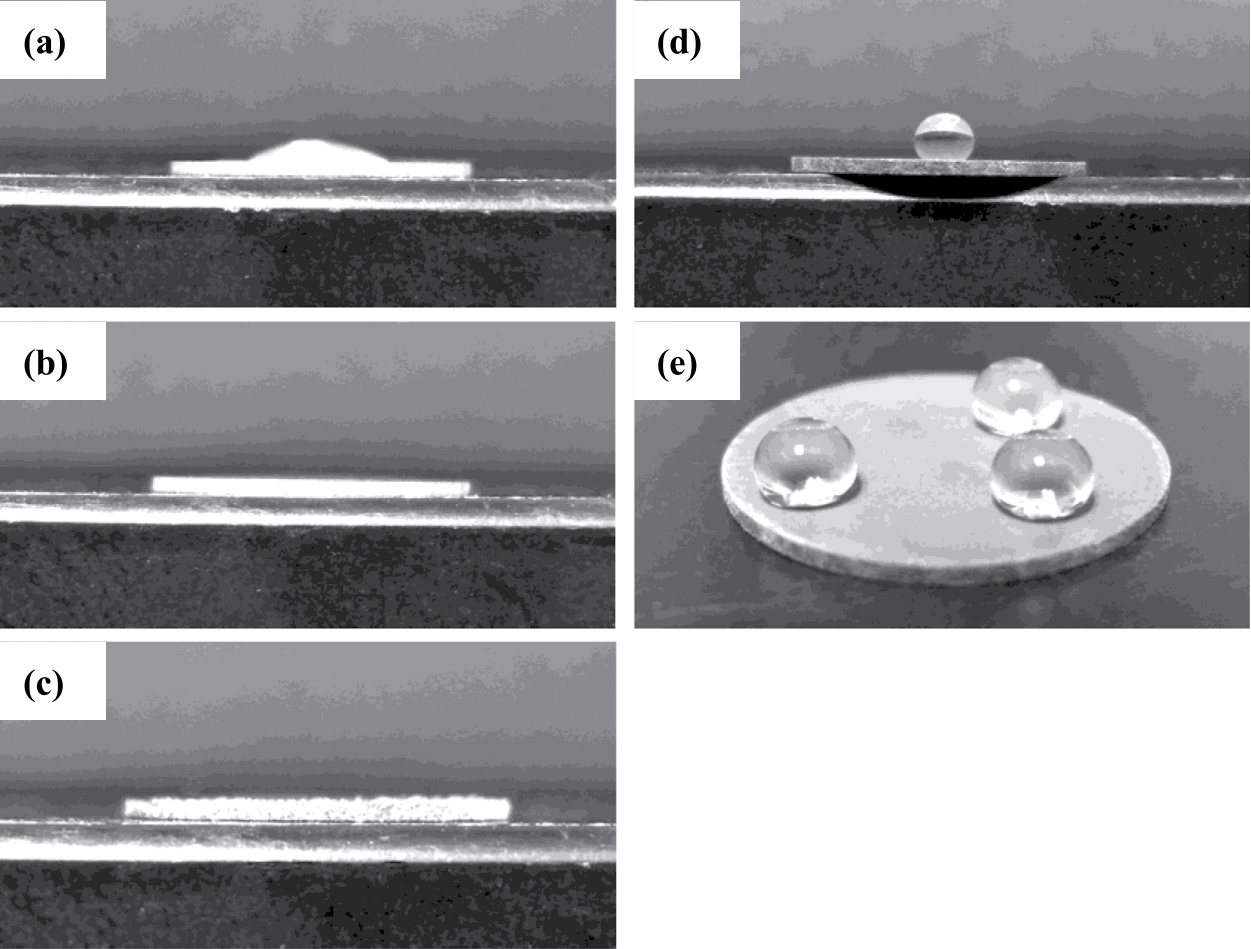
Hydrophilicity of the (**a**) uncoated AZ61 Mg alloy, (**b**) AZ61-AP, (**c**) AP-HT and (**d**) HT-SAM. (**e**) Multi-points contact angle of the water droplets on the HT-SAM.

Since wettability is an important property that is governed by both chemical composition and surface structure, the control of surface wettability directly affects the biocompatibility of materials in a physiological environment. Figure 3 shows images of water droplets on the surface of uncoated AZ61, alkaline pre-treated AZ61-AP, hydrothermally treated AP-HT and SAM-treated HT-SAM specimens by the static contact angle assessment. The contact angle of water droplets on the polished AZ61 Mg alloy is about 22°, as illustrated in Fig. 3(a). It is noted that the contact angles of water droplets on AZ61-AP and AP-HT surfaces are significantly less than 5°, as shown in Fig. 3(b) and (c). Referring to the XRD analysis results represented in Fig. 2(b) and (c), brucite Mg(OH)_2_ is the main phase composition of coatings on the surface treated AZ61 Mg alloy (i.e., AZ61-AP and AP-HT specimens). Since polar hydroxyl groups (OH^–^) can help to induce an attractive interaction between the Mg(OH)_2_ surface layers and water molecules through the hydrogen bond, it can be recognized that AZ61-AP and AP-HT specimens display a typical hydrophilic surface. Meanwhile, hydrothermally treated AP-HT coating also show a characteristic surface morphology with nanoscaled platelet-like crystals of brucite Mg(OH)_2_ (see Fig. 1(c)), and it can further improve the hydrophilicity of AP-HT specimens resulted from the capillary action of a rough surface morphology^43^. Therefore, the decrease in contact angle indicates that alkaline and hydrothermal treatments significantly improve the hydrophilicity of the AZ61 Mg alloy. Thereby cells adhesion and subsequent activities of cells will be further enhanced on these hydrophilic surfaces with nanoscaled platelet-like compounds^28,33,44^. Fig. 3(d) and (e) illustrate images of water droplets on the surface of HT-SAM specimens. The HT-SAM surface morphology almost show the same microstructural feature as the AP-HT (see Fig. 1(c) cf. (d) for the SEM surface morphologies), however, the contact angle of water droplets on HT-SAM specimens is about 140°, as illustrated in Fig. 3(d). The HT-SAM specimens obviously show a hydrophobic surface compared with the AP-HT specimens, and it can be deduced that the lack of surface wettability for HT-SAM specimens is resulted from the hydrophobic terminal methyl groups (–CH_3_) on the surface of AP-HT specimens after performing the SAM surface treatment by the T-BAG method.

**Figure 4:**
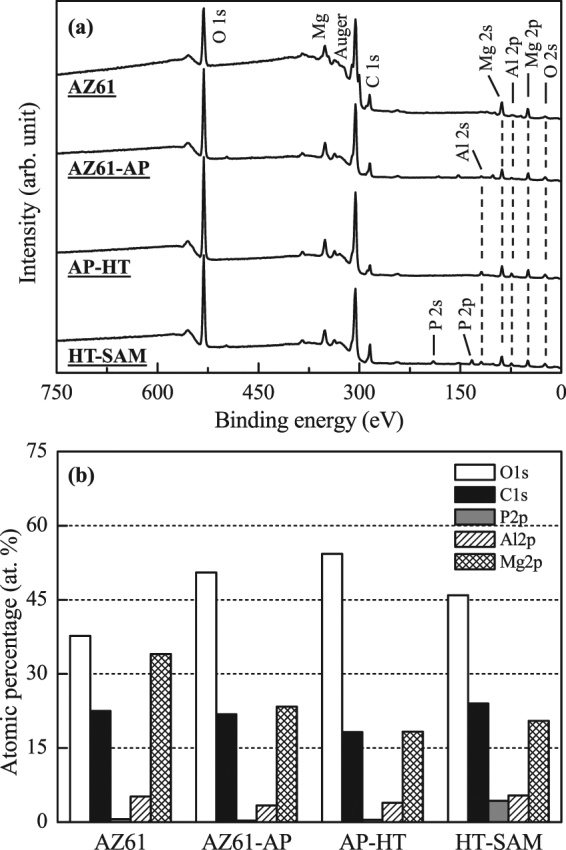
(**a**) XPS analysis results of the survey spectra and (**b**) the atomic percentage (at.%) of surface elements as detected by XPS for the AZ61 Mg alloy, AZ61-AP, AP-HT and HT-SAM.

The chemical states of AZ61 Mg alloy with various surface treatments are determined by XPS analysis and discussed in the following section. The signals of C, O, Mg, Al and P elements are detected from the survey XPS spectra in Fig. 4(a) and (b) shows the atomic percentage and makes a comparison of these detected surface elements for the AZ61 Mg alloy, AZ61-AP, AP-HT and HT-SAM specimens. Through the quantitative analysis of XPS results, the O/Mg, C/Mg and P/Mg molar ratios of various surface conditions are listed in Table 1. We can see that the atomic percentage of surface O element is significantly increased from about 38% to 55%, and the O/Mg ratio is also increased from 1.11 to 2.98. The increased oxygen content in AZ61-AP and AP-HT specimens is resulted from the Mg(OH)_2_ surface layer after applying alkaline and hydrothermal treatments to the AZ61 Mg alloy (see Fig. 2(b) and (c) for the XRD analysis). It is worth noting the surface P element (about 4.3 at.%, evaluated from the XPS spectrum in Fig. 4(a)) and P/Mg ratio of the HT-SAM is significantly higher than that of other surface treated conditions. The source of phosphorus content on the HT-SAM surface is resulted from the 1-butylphosphonic acid (C_4_H_9_PO(OH)_2_) during performing the SAM surface treatment.

**Table 1:** Comparison of surface O/Mg, C/Mg and P/Mg atomic ratios for the AZ61 Mg alloy, surface modified AZ61-AP, AP-HT, and HT-SAM specimens.

	O/Mg	C/Mg	P/Mg
AZ61	1.11	0.66	0.02
AZ61-AP	2.12	0.93	0.01
AP-HT	2.98	0.99	0.03
HT-SAM	2.24	1.17	0.21

**Figure 5:**
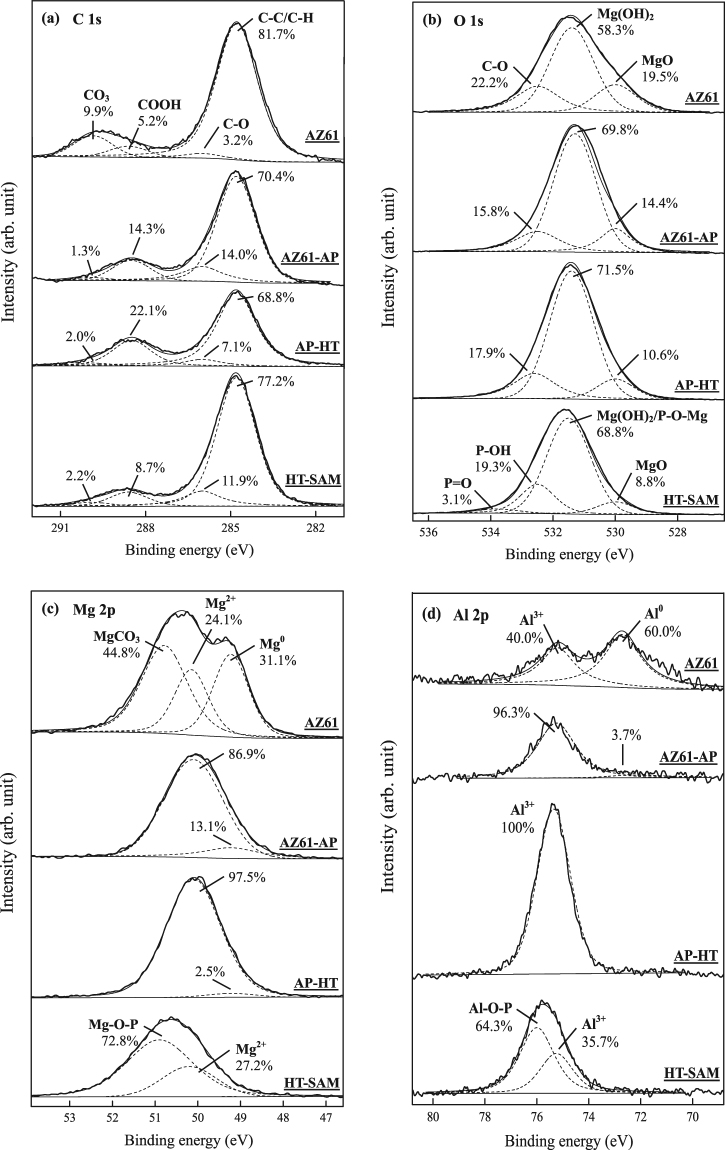
Effect of various surface treatments on the high-resolution XPS spectra of (**a**) C 1*s*, (**b**) O 1*s*, (**c**) Mg 2*p* and (**d**) Al 2*p* regions with curve-fitting analysis results.

Figure 5 shows the high resolution C 1*s*, O 1*s*, Mg 2*p* and Al 2*p* XPS spectra. Figure 5(a) shows a representative high-resolution C 1*s* band and curve-fitting results of the polished AZ61 Mg alloy, AZ61-AP, AP-HT and HT-SAM specimens. The maximum of C 1*s* peak located at binding energy (BE) of 284.8 eV is taken as a reference. The decreasing integration area of C-C/C-H bonding peak from 81.7% to 68.8% is resulted from a degreased surface of the AZ61 Mg alloy after the alkaline pre-treatment and the following hydrothermal treatment. In addition, the integration area of C-C/C-H bonding peak as well as the C/Mg ratio of the HT-SAM (see Fig. 5(a) cf. Fig. 4(b) and Table 1) is significantly increased with the directly chemical bonding of an organic alkyl chains monolayer on the HT-SAM surface by the T-BAG SAM treatment. The corresponding O 1*s* band of the AZ61, AZ61-AP and AP-HT specimens presented in Fig. 5(b) consists of three components at BE = 530.0 eV, BE = 531.5 eV and BE = 532.5 eV, which correspond to the native magnesium oxide (MgO), hydroxyl groups of the magnesium hydroxide (Mg(OH)_2_) and the C-O bonding, respectively. The decreasing integration area of native oxide (from 19.5% to 10.6%) and C-O chemical bonding (from 22.2% to 17.9%) are also resulted from a degreased surface of the AZ61 Mg alloy after the alkaline and hydrothermal treatments. Comparing Fig. 5(b) with Fig. 2(c), the significant increasing integration area of hydroxyl groups (from 58.3% to 71.5%) is resulted from the formation of Mg(OH)_2_ surface layer after the hydrothermal treatment. Considering the HT-SAM specimens, the deconvoluted peaks located at BE = 533.9 eV, BE = 532.4 eV and BE = 531.6 eV represent the presence of P=O double bond, P-OH and P-O-Mg bonds, respectively^40,45^. The P=O and P-OH bonds are highly polar head groups of the 1-butylphosphonic acid (C_4_H_9_PO(OH)_2_). The hydrophilic polar P-OH head groups will adsorb on the Mg(OH)_2_ during the T-BAG SAM treatment, and the reacted P-O-Mg covalent bond can be attributed to the dehydration of surface Mg(OH)_2_ with P-OH groups. Therefore, the XPS analysis results of C 1*s* and O 1*s* spectra demonstrate that a self-assembled monolayer (SAM) of the organic 1-butylphosphonic acid molecules can be directly bonded to the nanoscaled platelet-like Mg(OH)_2_ (i.e., the surface layer of hydrothermally treated AZ61 Mg alloy) by the T-BAG method.

Representative high resolution Mg 2*p* and Al 2*p* bands of various surface-treated specimens are shown in Fig. 5(c) and (d), respectively. Considering the untreated AZ61 Mg alloy, the Mg 2*p* composite peak is deconvoluted into three peaks at about BE = 49.3 eV, BE = 50.3 eV and BE = 51.0 eV corresponding to metallic magnesium (Mg^0^), Mg^2+^ ions in hydroxide (Mg(OH)_2_) and Mg^2+^ ions in carbonate (MgCO_3_), respectively^46^. A deconvoluted Al 2*p* peak at about BE = 72.7 eV corresponds to metallic aluminum (Al^0^), while an Al 2*p* peak at about BE = 75.3 eV with a minor intensity corresponds to the co-existence of Al^3+^ in oxide and hydroxide^46^. After the alkaline and hydrothermal surface treatments on AZ61 Mg alloy, we can see high resolution Mg 2*p* and Al 2*p* bands of AZ61-AP and AP-HT conditions only consist of a single peak corresponds to Mg^2+^ in Mg(OH)_2_ and Al^3+^ in Mg_2_Al(OH)_7_. It can be recognized as the formation of Mg(OH)_2_ and Mg_2_Al(OH)_7_ within the surface layer (see Fig. 5(c) and (d) cf. Fig. 2). In addition, the increasing peaks intensity of Mg 2*p* and Al 2*p* bands for AP-HT specimens can be attributed to the chemical stability of Mg(OH)_2_ and Mg_2_Al(OH)_7_ compounds is increased with performing the hydrothermal treatment. Considering the HT-SAM specimens, the deconvoluted Mg-O-P bond (at BE = 50.9 eV) in Mg 2*p* band and Al-O-P bond (BE = 76.0 eV) in Al 2*p* band demonstrate that the hydrothermally treated surface layer consists of Mg(OH)_2_ and Mg_2_Al(OH)_7_ compounds, and both of them can be bonded with hydrophilic polar P-OH head groups of the organic 1-butylphosphonic acid molecules after the SAM treatment.

**Figure 6:**
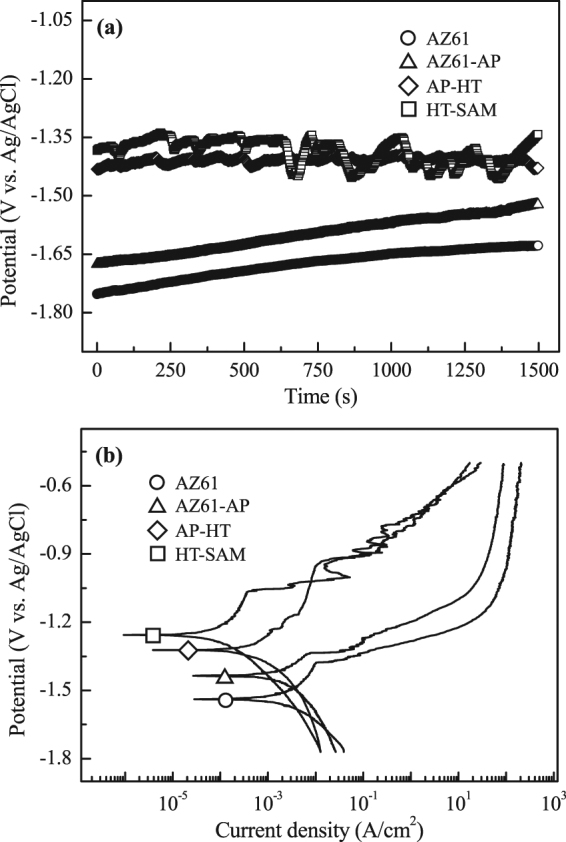
(**a**) OCP curves and (**b**) potentiodynamic polarization curves of the AZ61 Mg alloy, AZ61-AP, AP-HT and HT-SAM specimens tested in R-SBF solution at 37 °C.

Figure 6(a) plots the open-circuit potential (OCP) curves of the uncoated AZ61 Mg alloy, alkaline pre-treated AZ61-AP, hydrothermally treated AP-HT and SAM-treated HT-SAM specimens in the R-SBF solution. The OCPs of AZ61, AZ61-AP and AP-HT specimens are −1.67 V, −1.50 V and −1.41 V (vs. Ag/AgCl), respectively. The uncoated AZ61 Mg alloy has the most negative OCP value, and the OCP of AZ61 is improved after the alkaline pre-treatment. In addition, we can see the OCPs of AZ61 and AZ61-AP gradually increased during the measuring duration (1500 s). It means that the surface of AZ61 and AZ61-AP continues reacting with surrounding R-SBF solution. However, the OCP of AP-HT is increased and stably maintains at −1.41 V, which can be attributed to the effective protection of Mg(OH)_2_ surface layer against R-SBF solution after performing the hydrothermal treatment. The OCP of HT-SAM is slightly higher than that of AP-HT. Meanwhile, the OCP fluctuates between −1.33 V and −1.45 V, and the data fluctuation of OCP is attributed to the hydrophobic surface of HT-SAM specimens. The results reveal that a significant surface passivation of the AZ61 Mg alloy can be achieved by the hydrothermal and following SAM treatments.

Potentiodynamic polarization curve can provide powerful information on the corrosion behaviors and corrosion rate. Figure 6(b) shows the corrosion test results in terms of the potentiodynamic polarization curves of the uncoated AZ61 Mg alloy and the three surface treated Mg specimens in the R-SBF solution at 37 °C. The corrosion potential (*E*_corr._) and the corrosion current density (*I*_corr._) are calculated from the polarization curves using the Tafel extrapolation method. The polarization resistance (*R*_p_), which is inversely proportional to the corrosion current density, is calculated from Eq. (1). The parameters *β*_a_ and *β*_c_ are the anodic Tafel slope and cathodic Tafel slope of the polarization curves, respectively. The corresponding corrosion rate (*P*_i_) is evaluated according to the relationship *P*_i_ = 22.85 × *I*_corr_.

1 $${R}_{p}=\frac{{\beta }_{a}{\beta }_{c}}{2.3\cdot ({\beta }_{a}+{\beta }_{c})\cdot {I}_{corr.}}$$

Table 2 records the corrosion parameters obtained from corrosion tests in R-SBF. As shown in Fig. 6(b) and Table 2, it is observed that the uncoated AZ61 Mg alloy exhibits the most negative corrosion potential (−1.54 V) of all the conditions. The corrosion potential of AZ61-AP slightly shifts to −1.44 V, and the polarization resistance of the AZ61 Mg alloy (54.3 Ω-cm^2^) is also increased with the improvement of corrosion resistance by the alkaline pre-treatment (AZ61-AP, *R*_p_ = 267.0 Ω-cm^2^). It is noted that the corrosion potential is significantly improved to −1.32 V for the hydrothermally treated AP-HT and −1.26 V for the SAM-treated HT-SAM specimens. Meanwhile, the corrosion current density is dramatically decreased from 28.4 μA/cm^2^ of the uncoated AZ61 alloy to 3.9 μA/cm^2^ and 0.9 μA/cm^2^ (Table 2) of the AP-HT and HT-SAM, respectively. The HT-SAM specimens display the highest corrosion potential and the lowest corrosion current density of all the conditions.

**Table 2:** Electrochemical parameters of the AZ61 Mg alloy, surface modified AZ61-AP, AP-HT, and HT-SAM specimens in R-SBF at 37 °C.

	corrosion potential *E*_corr._ (V vs. Ag/AgCl)	corrosion current density *I*_corr._ (μA/cm^2^)	polarization resistance *R*_p_ (Ω-cm^2^)	corrosion rate *P*_i_ (μm/year)
AZ61	−1.54	28.4	54.3	684.9
AZ61-AP	−1.44	26.9	267.0	614.7
AP-HT	−1.32	3.9	1165.6	88.0
HT-SAM	−1.26	0.9	967.7	21.3

In general, a shift of the corrosion potential toward the positive side and a decrease of the corrosion current density mean that the corrosion reaction of a metallic substrate is significantly suppressed by the surface treatment. Although the corrosion potential of AZ61-AP seems to be higher than that of uncoated AZ61 Mg alloy, a little difference in the corrosion current density between the AZ61 (28.4 μA/cm^2^) and the AZ61-AP (26.9 μA/cm^2^) specimens indicates that a thin surface hydroxide layer (Mg(OH)_2_) obtained from the alkaline pre-treatment is insufficient to produce a desirable corrosion resistance to the AZ61 Mg alloy in R-SBF. Referring to the observation of surface morphologies, it can be recognized that the formation of thick nanoscaled platelet-like Mg(OH)_2_ on hydrothermally treated AP-HT specimens (see Fig. 1(c)) can provide an effective protectiveness to prevent the penetration and direct contact of R-SBF solution because of the apparently higher corrosion potential and lower corrosion current density of the AP-HT specimens. Also, we can see the polarization resistance (*R*_p_) of the AZ61 Mg alloy is increased with the improvement of corrosion resistance by performing various surface treatments (Table 2). The polarization resistance of AP-HT and HT-SAM specimens is 1165.6 and 967.7 Ω-cm^2^, respectively. These values are significantly larger than that of the uncoated AZ61 Mg alloy (54.3 Ω-cm^2^) and the alkaline pre-treated AZ61-AP specimens (267.0 Ω-cm^2^). Since the enhancement of the polarization resistance is related to the formation of a surface protective barrier against the corrosive environment for the substrate, the presence of either hydrothermally synthesized Mg(OH)_2_ coatings, or the deposition of hydrophobic organic surface self-assembled monolayer, can lead to a substantial decrease in the corrosion rate of AZ61. In other words, both of the hydrothermal and T-BAG SAM surface treatments applied in this study significantly enhance the corrosion resistance and improve all corrosion parameters of AZ61 Mg alloy in R-SBF.

**Figure 7:**
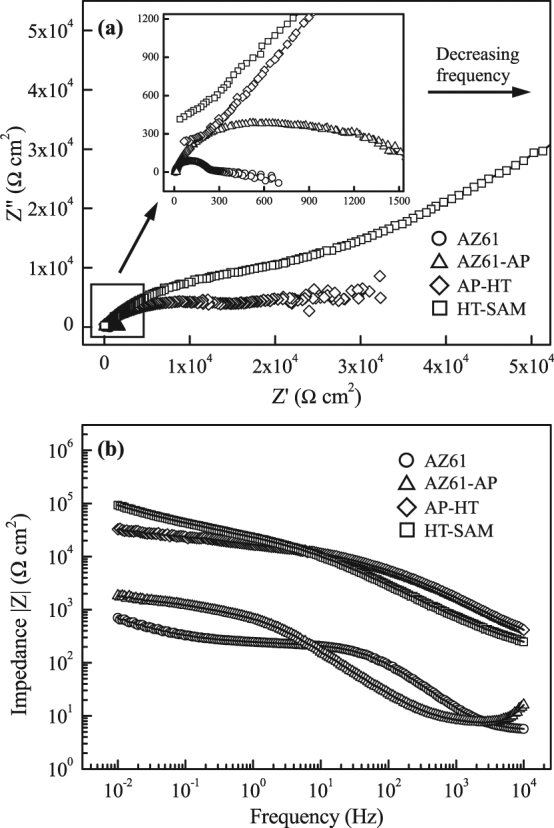
(**a**) Nyquist plots and (**b**) Bode plots of the impedance magnitude of the AZ61 Mg alloy, AZ61-AP, AP-HT and HT-SAM specimens tested in R-SBF at 37 °C.

The corrosion protective nature of various surface treatments is further analyzed using electrochemical impedance spectroscopy (EIS) in this study. Figure 7 shows the Nyquist and Bode plots of the uncoated AZ61 Mg alloy, alkaline pre-treated AZ61-AP, hydrothermally treated AP-HT and SAM-treated HT-SAM specimens. As seen in the Nyquist plot (Fig. 7(a)), the impedance increases with decrease in the frequency. The real impedance (Z′) and imaginary impedance (Z″) of AP-HT and HT-SAM specimens are much higher than that of uncoated AZ61 and AZ61-AP specimens. In addition, the diameter of the capacitive loop in the high frequency range represented the charge transfer resistance (*R*_ct_) related to the dynamic corrosion behaviors, and the *R*_ct_ value is inversely proportional to the electrochemical corrosion reaction rate at the substrate/electrolyte interface. The corresponding *R*_ct_ values of uncoated AZ61 Mg alloy, AZ61-AP, AP-HT and HT-SAM specimens calculated from the Nyquist plot (Fig. 7(a)) are about 0.24, 1.11, 18.05 and 36.04 kΩ-cm^2^, respectively. As seen in the Bode plot (Fig. 7(b)), the impedance magnitude (|Z|) of these specimens decreases with increasing the frequency, and the impedance of surface treated specimens, especially for the AP-HT and HT-SAM specimens, is also significantly higher than that of uncoated AZ61 Mg alloy. In general, the higher corrosion potential (*E*_corr._), charge transfer resistance (i.e., polarization resistance) and the lower corrosion current density (*I*_corr._) indicate that the specimen has better corrosion resistance. Referring to the above-mentioned corrosion parameters (see Table 2 cf. Fig. 7(a) and (b)), the results confirm that the hydrothermally synthesized Mg(OH)_2_ (AP-HT) and the deposition of hydrophobic organic surface self-assembled monolayer (HT-SAM) can effectively reduce the charge transfer process at the interface between AZ61 Mg alloy and the corrosive R-SBF solution.

Moreover, the Nyquist plot of HT-SAM specimens has a linear correlation between Z′ and Z″ in the low frequency range (as illustrated in Fig. 7(a)), and this additional resistive element can be regarded as the Warburg impedance (W). Warburg impedance is generally induced by the diffusion-controlled mechanisms of corrosive reactants or electrolyte ions in a surface coating, and the Warburg behavior is often observed for a passive layer with a high charge transfer resistance (*R*_ct_). It is reported that a passive conversion coating, which exhibits a low corrosion current density, a high charge transfer resistance and Warburg impedance, can effectively protect Mg alloy from corrosion^47^. Comparing the data of AP-HT and HT-SAM specimens as represented in Fig. 7, the higher *R*_ct_ value and the presence of Warburg impedance to HT-SAM specimens demonstrate that the hydrophobic self-assembled monolayer (SAM) served as a passive surface layer with good dielectric properties and effective charge transfer resistance to the nanoscaled platelet-like Mg(OH)_2_ coated AZ61 Mg alloy in R-SBF. The results of EIS tests is in agreement with the potentiodynamic polarization measurements, which indicating a preferable corrosion resistance can be achieved by the hydrothermal and SAM surface treatments.

**Figure 8:**
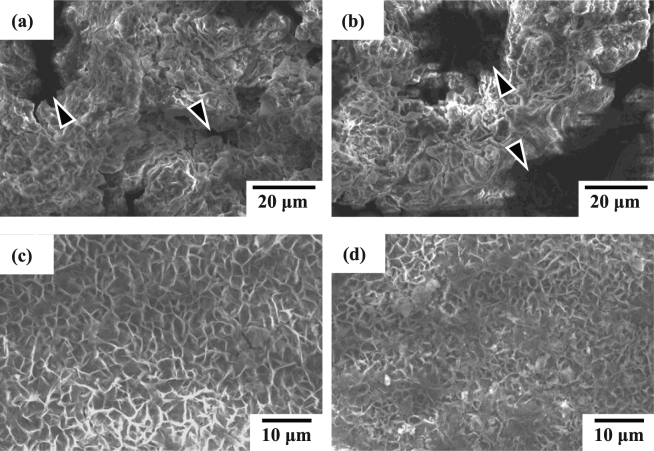
SEM surface morphologies of the (**a**) AZ61 Mg alloy, (**b**) AZ61-AP, (**c**) AP-HT, and (**d**) HT-SAM after potentiodynamic polarization tests.

Figure 8 shows the SEM micrographs of sample surface after potentiodynamic polarization experiments. Figures 8(a) and (b) illustrate the corroded surface morphologies of uncoated AZ61 Mg alloy and alkaline pre-treated AZ61-AP specimens, respectively. We can see the AZ61-AP specimens show a corroded surface similar to the AZ61 Mg alloy. Moreover, an apparent pitting corrosion can be observed on the corroded surface of both AZ61 and AZ61-AP specimens as those indicated by the triangular marks in Fig. 8(a) and (b). When corrosive solution contains chloride ions (Cl^−^), Mg^2+^ generated in the anodic reaction will react with Cl^−^ and severe corrosion occurs for Cl^−^ concentrations exceeding 30 mmol/L in physiological environments^5^. Therefore, it can be recognized that the pitting corrosion of AZ61 and AZ61-AP specimens is resulted from the high Cl^–^ concentration of R-SBF solution. The anti-corrosion effect is obviously insufficient for Mg alloys only with the alkaline pre-treatment. Figure 8(c) shows the corroded surface morphology of hydrothermally treated AP-HT specimens for illustration. Comparing Fig. 8(c) with Fig. 1(c), we can see less variation and no significant pitting corrosion are observed on the corroded AP-HT surface. According to the phase composition analysis (see Fig. 2(c)) and electrochemical testing results (see Table 2 and Fig. 6), nanoscaled platelet-like Mg(OH)_2_ coated AP-HT specimens display lower corrosion current density and better corrosion resistance. It confirms that the hydrothermal surface treatment is available and the Mg(OH)_2_ coating is useful of corrosion protection for Mg alloys. Figure 8(d) shows the corroded surface morphology of T-BAG SAM-treated HT-SAM specimens for illustration. It can be seen that the hydrophobic self-assembled monolayer modified hydrothermal Mg(OH)_2_ coating still possesses structural integrity without pitting corrosion after electrochemical tests. Referring to the XPS analysis and EIS measurements as-mentioned in Figs 5 and 7, it demonstrates that a significant chemical bonding between the organic self-assembled monolayer and Mg(OH)_2_ can further enhance the surface chemical stability and corrosion resistance of Mg alloys in physiological environments.

## Conclusion

Through the hydrothermal treatment, a uniform nanoscaled platelet-like crystalline Mg(OH)_2_ coating with minor Mg/Al hydrotalcite Mg_2_Al(OH)_7_ phase can be easily produced on the AZ61 Mg alloy. XPS analysis demonstrates that an organic and hydrophobic 1-butylphosphonic acid self-assembled monolayer (SAM) can be directly bonded to the hydrothermally synthesized Mg(OH)_2_ without varying surface morphologies through the tethering by aggregation and growth (T-BAG) method. The formation of thin Mg(OH)_2_ only by the alkaline pre-treatment is insufficient to give a desirable corrosion resistance to Mg alloys. The hydrothermally synthesized Mg(OH)_2_ conversion coatings can further provide a substantial improvement in the corrosion resistance and a significant reduction in the corrosion rate of AZ61 Mg alloy in R-SBF solution. As a result of EIS measurements, higher polarization resistance and the presence of Warburg impedance to HT-SAM specimens demonstrate that the 1-butylphosphonic acid self-assembled monolayer by the T-BAG can serve as a passive surface layer with an effective charge transfer resistance further contributing to the anti-corrosion ability of hydrothermally synthesized Mg(OH)_2_ coated AZ61 in R-SBF solution.

## Methods

### Preparation of hydrothermal coating and SAM treatment by T-BAG method

The base metal used in this study was 0.6 mm-thick die-cast Mg-Al-Zn sheets with a chemical composition of 6.1 Al, 0.9 Zn, 0.2 Mn, 0.038 Si, and Mg balance (in wt.%, named AZ61), which was determined by inductively coupled plasma-atomic emission spectrometry (ICP-AES, PerkinElmer/Spectrum one). These sheets were then CNC machined into disc specimens with a diameter of 12.7 mm and thickness of 0.6 mm for the following surface treatments. Prior to hydrothermal coating process, AZ61 disc specimens were ground with 2000 grit SiC papers, carefully polished to 6000 grit, ultrasonically cleaned in acetone, absolute alcohol for 10 min, and dried in N_2_ gas. In order to degrease and stabilize the oxides into Mg conversion coatings, the cleaned AZ61 disc specimens were then alkaline pre-treated in a 10 M KOH solution at 70 °C, and they will be denoted by “AZ61-AP (alkaline pre-treatment)” in the following. The AZ61-AP specimens were also ultrasonically cleaned in absolute alcohol, and then dried in N_2_ gas.

The hydrothermal treatment of AZ61-AP specimens was performed at 125 °C and held for 24 h in an autoclave (Parr 4621). The autoclave contained 250 ml of deionized water, which was used as the source of saturated steam atmosphere during the hydrothermal treatment. The heating temperature was maintained throughout the experiments using a heater attached to the autoclave. The processing temperature was precisely controlled by a proportional-integral-derivative (PID) temperature controller (Parr 4842) with ± 1 °C. The hydrothermally treated specimens will be designated as “AP-HT (alkaline pre-treatment/hydrothermal treatment)”.

**Figure 9:**
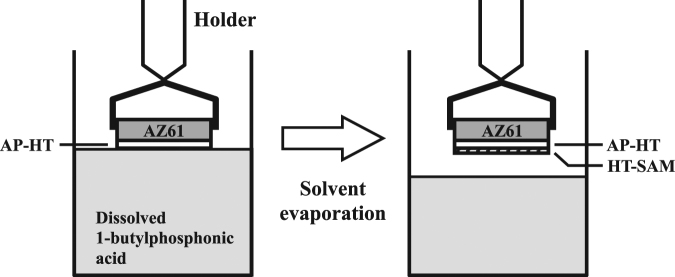
Schematic apparatus of the T-BAG method for the self-assembled monolayer (SAM) surface treatment of AP-HT specimens.

Analytical grade of 1-butylphosphonic acid (CH_3_CH_2_CH_2_CH_2_PO(OH)_2_ or C_4_H_11_O_3_P, purity 98%, Alfa Aesar) was used for preparing the self-assembled monolayer (SAM) bound directly to the surface of AP-HT specimens through the tethering by aggregation and growth (T-BAG) method^38^. Figure 9 shows a schematic apparatus of the T-BAG setup for the SAM surface treatment. The AP-HT specimens were held vertically and immersed in a solution of the 1-butylphosphonic acid (2 mM in absolute alcohol) below its critical micelle concentration (CMC) in a beaker. The solvent was allowed to evaporate slowly for 7 h, until the level of the solution fell below the substrates. As the meniscus traverses the substrate, the 1-butylphosphonic acid was then transferred to the surface of AP-HT specimens. The surface treated AP-HT specimen was removed from its holder, ultrasonically cleaned in absolute alcohol, and was heated at 120 °C in a vacuum oven for 12 h to bond the SAM to the surface of AP-HT specimen. Finally, any multilayer was removed by 0.2 M Na_2_CO_3_ solution, and then dried in N_2_ gas. These SAM surface treated specimens will be designated as “HT-SAM (alkaline pre-treatment/hydrothermal treatment/SAM by T-BAG method)”.

### Microstructure characterization

The phase compositions of AZ61, alkaline pre-treated (AZ61-AP), hydrothermally treated (AP-HT) and SAM surface treated by T-BAG (HT-SAM) samples were identified by a thin film X-ray diffractometer (D8 DISCOVER Bruker AXS Gmbh, Karlsruhe, Germany), using Cu Kα radiation at 40 kV and 40 mA over a 2θ range of 15–80° with a scan speed of 2° (2θ) min^–1^ (step size, 0.02°). The chemical states of polished AZ61 alloy, surface treated AZ61-AP, AP-HT and HT-SAM samples were determined by X-ray photoelectron spectroscopy (XPS, VGS Thermo K-Alpha) using a standard Al Kα radiation (1486.7 eV) at a pressure of 3 × 10^–9^ torr. As required, the measured binding energy (BE) scale was referenced to the adventitious C 1*s* at the BE of 284.8 eV. For each specimen, survey spectra and high-resolution spectra of the C 1*s*, O 1*s*, Mg 2*p* and Al 2*p* regions were obtained. The Gaussian peak-fitting routine was used in the analysis of high-resolution spectra for separating species in different chemical states. Changes in the surface morphologies and compositions of the AZ61-AP, AP-HT and HT-SAM samples were characterized by low/variable vacuum scanning electron microscopy (LV-SEM, JSM-6390LV), equipped with an energy-dispersive X-ray spectrometer attachment (EDS, OXFORD/INCA 350). The static contact angle assessment was applied for wettability studies of the AZ61 Mg alloy with various surface treatments. All the samples were cleaned with absolute alcohol, dried in N_2_ gas and the contact angles were measured using 10 μl distilled water as the solvent.

### Electrochemical corrosion behavior tests

The electrochemical properties of AZ61, AZ61-AP, AP-HT and HT-SAM specimens were conducted by a Princeton Applied Research PARSTAT 2273 potentiostat. The corrosion cell used in this study was a three-electrode assembly, in which platinum sheet and Ag/AgCl were used as the counter electrode and the reference electrode, respectively. AZ61 alloy and different surface treated specimens were applied as the working electrode. The standard simulated body fluid (R-SBF) with a pH of 7.4 was prepared according to the Kokubo and Takadama’s recipe^48^ in which the ions concentrations were similar to the human blood plasma, as listed in Table 3.

**Table 3:** Nominal ion concentrations (mM) of the used standard simulated body fluid (R-SBF).

	Na^+^	K^+^	Mg^2+^	Ca^2+^	Cl^−^	HCO_3_^−^	HPO_4_^2−^	SO_4_^2−^
R-SBF	142.0	5.0	1.5	2.5	147.8	4.2	1.0	0.5
human blood plasma	142.0	5.0	1.5	2.5	103.0	27.0	1.0	0.5

The open-circuit potential (OCP) was measured in the corrosion cell that contained standard R-SBF solution. The measuring duration was 1500 s. Electrochemical impedance spectroscopy (EIS) measurements were performed from 10^5^ to 10^–1^ Hz, and the AC amplitude of the sinusoidal voltage signal was 10 mV. Prior to starting of the experiments, the specimens were immersed into the R-SBF solution for 30 min to stabilize the open-circuit potential. The EIS data measured at the established open-circuit potential were fitted with the Powersuite software, and the Nyquist and Bode plots were obtained from the analysis of the impedance data. The corrosion behaviors of AZ61, AZ61-AP, AP-HT and HT-SAM specimens within R-SBF at 37 °C were investigated by potentiodynamic polarization tests according to the ASTM G102-89. A three-electrode cell was used for potentiodynamic polarization tests. All experiments were carried out in the R-SBF at a constant scan rate of 0.5 mV/s. The anodic and cathodic polarization curves were obtained for each specimen. Corrosion current densities and corrosion potentials were determined from the potentiodynamic polarization curves by Tafel extrapolation methods.
